# Combination of 9-aminoacridine with Campath-1H provides effective therapy for a murine model of adult T-cell leukemia

**DOI:** 10.1186/1742-4690-11-43

**Published:** 2014-06-02

**Authors:** Wei Ju, Meili Zhang, Michael Petrus, Michiyuki Maeda, Cynthia A Pise-Masison, Thomas A Waldmann

**Affiliations:** 1 Lymphoid Malignancies Branch, Center for Cancer Research, National Cancer Institute, NIH, 10 Center Drive, Building 10, Room 4 N115, Bethesda, MD 20892-1374, USA; 2 Institute for Virus Research, Kyoto University, Kyoto, Japan; 3 Vaccine Branch, Center for Cancer Research, National Cancer Institute, NIH, Building 41, Room C303, 9000 Rockville Pike, Bethesda, MD 20892, USA

**Keywords:** HTLV-1, ATL, p53, NF-κB

## Abstract

**Background:**

Adult T-cell leukemia/lymphoma (ATL) is an aggressive malignancy of CD4^+^CD25^+^ lymphocytes caused by human T-cell lymphotropic virus type 1. While much progress has been made in understanding the mechanisms of cellular dysregulation, the prognosis for aggressive ATL still remains poor. Therefore, new therapeutic approaches need to be developed.

**Results:**

Previously, we demonstrated that the viral protein Tax inactivates p53 in HTLV-1-infected T-cells. Here we show that 9-aminoacridine (9AA) through p53 reactivation and NF-κB inhibition has selective toxicity for infected leukemic cells independent of their p53 status. We further demonstrate that 9AA activates caspase-3/7 resulting in PARP cleavage. Next we investigated the efficacy of 9AA in the MET-1 ATL model. Alone, 9AA did not cause significant drops in surrogate tumor markers, soluble IL-2Rα or β2-micorglobulin (β2μ) levels with only a slight increase in survival of MET-1-bearing mice. However, in combination with Campath-1H, 9AA treatment resulted in low soluble IL-2Rα and β2μ levels at 2 and 4 weeks. Consistent with reduced tumor cell burden, combination treatment significantly increased survival of MET-1-bearing mice compared to mice treated with either drug alone. Splenic cells isolated from 9AA or combination treated mice showed increased p53 protein levels and transcriptional activity. Consistent with increased tumor suppressor activity, we found increased PARP-1 cleavage in 9AA and combination treated cells.

**Conclusion:**

Our results indicate that targeting reactivation of p53 and inhibition of NF-κB with acridine-derivatives in combination with other chemotherapeutics could result in increased efficacy and selective killing of tumor cells.

## Background

The human T-lymphotropic virus type 1 (HTLV-1) is the causative agent of adult T-cell leukemia/lymphoma (ATL). ATL is an aggressive lymphoid proliferative disease which is characterized by the monoclonal or oligoclonal integration of HTLV-1 provirus in the tumor cells [1-3]. Typical ATL cells are characterized by unusual morphology with a lobulated nucleus, known as “flower cells” [4]. These malignant lymphocytes are activated CD4+ CD25+ T cells with increased expression of the alpha chain of the interleukin-2 receptor (CD25). ATL develops in a small percentage (2-4%) of HTLV-1-infected individuals after a long period of clinical latency following viral infection. The late onset of ATL indicates that HTLV-1 infection is not sufficient to induce T-cell transformation and that additional genetic mutations favoring the leukemogenic process are needed before the onset of ATL [5].

Several studies support the key role for the tumor suppressor p53 plays in cancer prevention (reviewed in [6] and [7]). The importance of p53 in protecting cells from malignant transformation is reflected by the high frequency of mutations of p53 found in human cancers [6]. It is estimated that 50% of human cancers have alterations in the p53 gene resulting in inactivation or loss of the protein [7,8]. Often, even in cancers retaining a wild-type p53 gene, it is functionally inactive [6]. Thus developing strategies for restoring p53 function in tumor cells is a promising approach to treating human cancers [7-10].

A number of investigators have shown that while the majority of patients with adult T- cell leukemia retain a wild-type p53, it is functionally inactive [11-16]. The viral oncoprotein, Tax has been shown to inhibit the transcriptional and cell cycle arrest functions of p53 [11,13,16]. Our group has shown that the ability of Tax to activate NF-κB correlates with p53 inhibition [17]. Further, Tax-mediated p53 inactivation involves the interaction of p53 with the p65 subunit of NF-κB and requires IKK-2 and AKT activation [18-21]. Indeed, treatment of HTLV-1 transformed cells with inhibitors of either IKK-2 or AKT restored p53 function [19-21].

Identified by Gurova and colleagues [22] in a screen of compounds to restore p53-dependent transactivation in a renal carcinoma cell line, 9-aminoacridine (9AA) is a drug structurally similar to the antimalaria drug quinacrine which is currently in clinical trials for recurrent pleural effusion caused by malignant tumors [23]. In RCC45 cells, 9AA was found to increase p53 transcription activity and to inhibit NF-κB activation [22]. In addition, we found that 9AA is effective at reactivating p53 in HTLV-1 transformed cells in an NF-κB/AKT dependent manner [24]. 9AA preferentially induced p53-dependent apoptosis in HTLV-1 infected cells C8166, MT2 and Hut102 compared to control peripheral blood mononuclear cells or uninfected T-cell lines.

In the present study, we investigated the effect of 9AA in cytokine-dependent and cytokine-independent true ATL leukemic cell lines. All HTLV-1 infected cell lines were selectively killed by 9AA independent of cytokine dependency or p53 status. We further tested the efficacy of 9AA in our xenograft ATL model when used alone or in combination with Campath-1H (humanized anti-CD52 antibody, alemtuzumab). MET-1-bearing mice showed minimal reduction in sIL-2Rα and β2-microglobulin (β2μ) levels when treated with 9AA alone. In contrast, mice treated with Campath-1H or Campath-1H plus 9AA showed marked reduction in both sIL-2Rα and β2μ. Importantly, Campath-1H plus 9AA significantly prolonged the survival of the mice compared to untreated, 9AA alone or Campath-1H alone treated mice. MET-1 cells showed increased p53 protein levels and transcription activity when treated with 9AA or the combination of 9AA plus Campath-1H. Consistent with increased tumor suppressor activity, we found increased PARP-1 cleavage in 9AA and 9AA plus Campath-1H treated cells.

## Results

### 9AA inhibited proliferation of HTLV-1-transformed cell lines and HTLV-1-infected ATL cells

**Figure 1:**
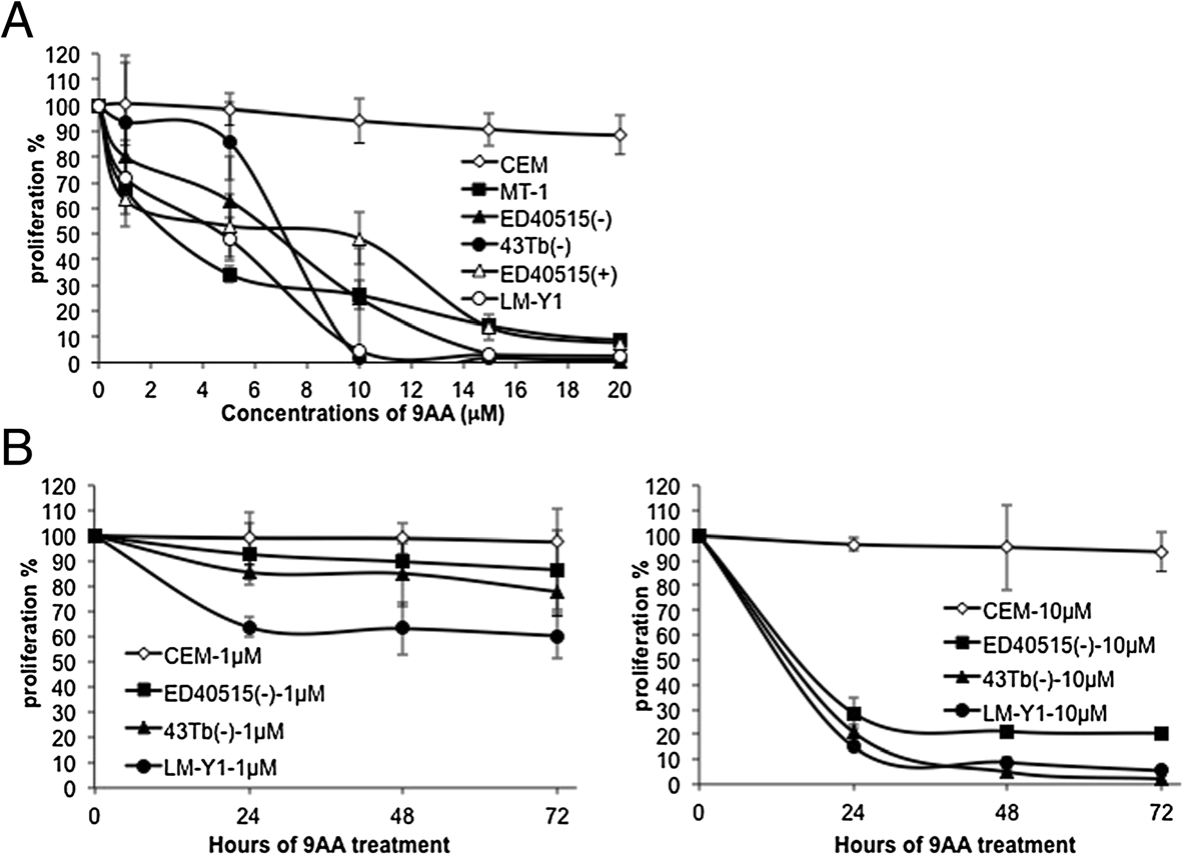
**9AA inhibited proliferation of HTLV-1-transformed cell lines and HTLV-1-infected ATL cells. (A)** The proliferation of HTLV-1-infected ATL cell lines was inhibited by 9AA. The HTLV-1-infected ATL cell lines MT-1, ED40515 (-), 43 Tb (-), ED40515 (+), and LM-Y1 were treated for 48 hours with and without serially increasing concentrations of 9AA. CEM cell line (an HTLV-1 negative, human T lymphoblastic leukemia cell line) was used as a control. Data are presented as means ± SD. **(B)** The proliferation of ATL cell lines was inhibited when treated with 9AA for different times. CEM, ED40515 (-), 43 Tb (-), and LM-Y1 cell lines were treated with and without 9AA at 1 μM (left panel), or 10 μM (right panel), for 24, 48, or 72 hours, respectively. Data are representative of 3 independent experiments.

We had previously shown that 9AA reduced the cell viability of HTLV-1 transformed cells carrying wild type p53 [24]. To determine if 9AA is also effective at reducing the viability of true ATL cell lines, we tested five HTLV-1 infected ATLs that are both IL-2 dependent and independent and carry wild type and mutant p53. HTLV-1 infected ATL cell lines MT-1, ED40515 (-), 43 Tb (-), ED40515 (+), and LM-Y1 and one HTLV-1 uninfected cell line, CEM were treated with or without increasing concentrations of 9AA in culture for 48 hours. 9AA inhibited proliferation of all five ATL cell lines regardless of cytokine dependence or p53 status in a concentration dependent manner. In contrast, the HTLV-1 negative, CEM cell line was only minimally affected by 9AA even at a concentration of 20 μM (Figure 1A). Treatment of ED40515 (-), 43 Tb (-), LM-Y1 and CEM cells with 9AA at a low concentration of 1 μM and a high concentration of 10 μM for 24, 48, or 72 hours resulted in a dose and time dependent decrease in cellular proliferation of the ATL lines (Figure 1B). Again, 9AA had little effect on the cellular proliferation of CEM cells.

### 9AA induced apoptosis in ATL cell lines

**Figure 2:**
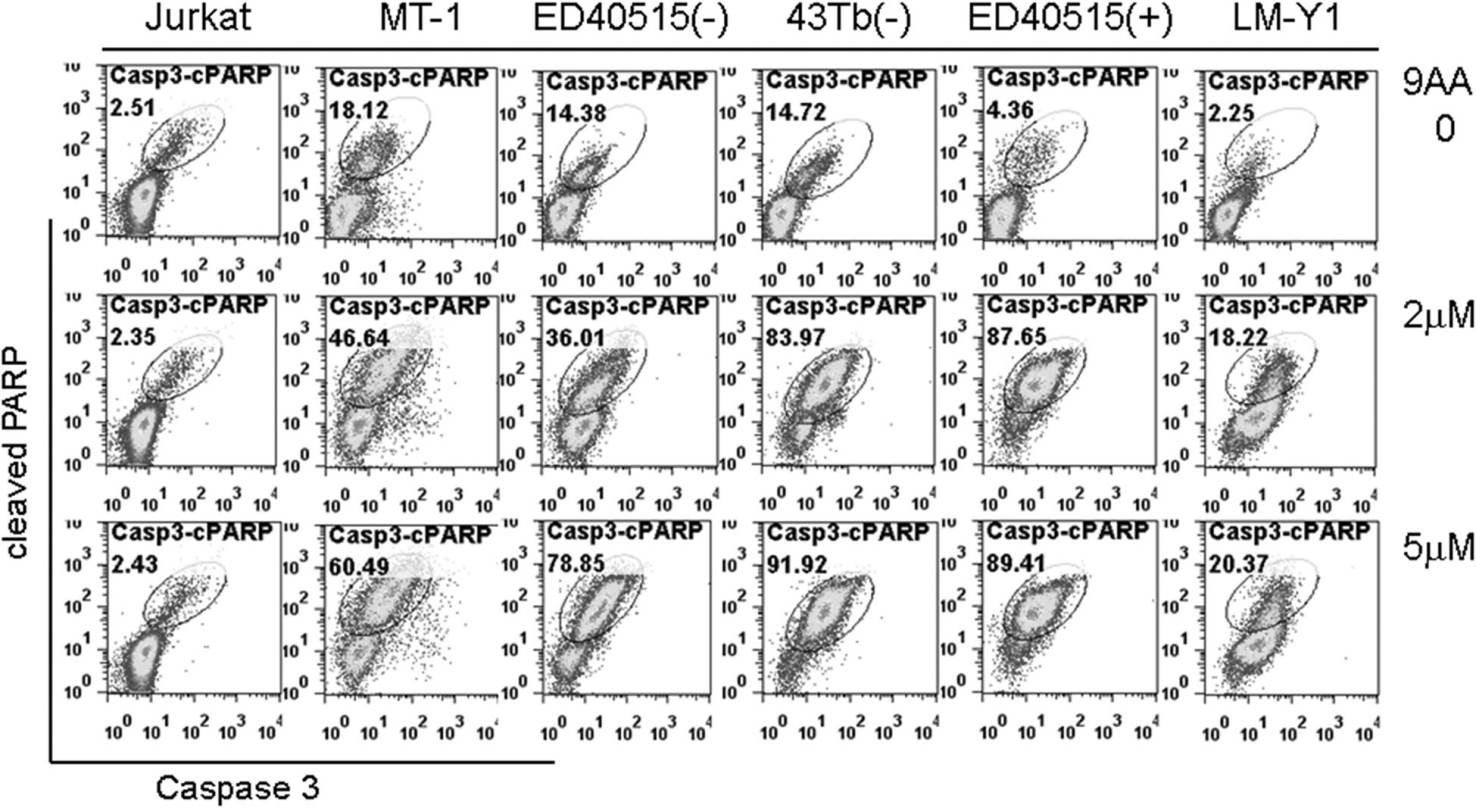
**Increased active caspase-3 and cleaved PARP in ATL cell lines after 9AA treatment.** Jurkat, MT-1, ED40515 (-), 43 Tb (-), ED40515 (+), and LM-Y1 cells were treated for 48 hours with and without 9AA at 2 μM, or 5 μM. The cells were stained with anti-cleaved PARP and anti-active caspase3, and then analyzed by FACS for apoptotic cells with active caspase-3 and cleaved PARP. Data are representative of 3 independent experiments.

**Figure 3:**
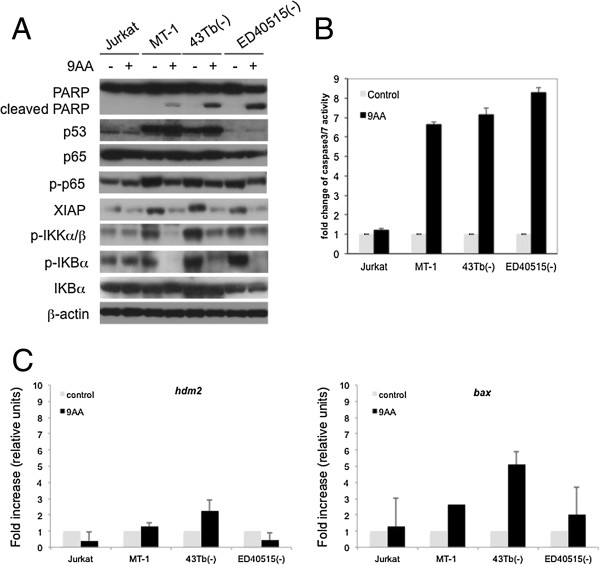
**9AA increased expression of p53 and activity of caspase-3/7, downregulated the NF-κB pathway, and induced apoptosis in ATL cell lines.** Jurkat, MT-1, ED40515 (-), and 43 Tb (-) cells were treated for 48 hours with and without 9AA at 5 μM. Data are representative of 3 independent experiments. **(A)** Western blot analysis of the expression levels of proteins in ATL cell lines before and after 9AA treatment. Cell lysates were immunoblotted with the indicated antibodies: PARP, p53, phospho-p65, p65, XIAP, and phosph-IKKα/β, phosphr-IκBα and IκBα. β-actin was used as an input control. **(B)** Detection of caspase-3/7 activity in ATL cell lines before and after 9AA treatment. Cells were measured for the activities of caspase-3/7. Fold change was calculated based on the activity of caspase-3/7 before treatment. Data are representative of 3 independent experiments and are presented as means ± SD. **(C)** Induction of p53 responsive genes in 9AA treated cells. Total cellular mRNA was extracted from cell lysates of 9AA (10 μM) or control cells 48 hours after treatment. Quantitative PCR was performed to measure the expression levels of *hdm2* (left panel) and *bax* (right panel) genes. RNA levels of control treated cells were set at 1. Each sample was run in triplicate from two independent experiments. The MT1 cell line was run in triplicate from one experiment. Expression levels of p53 responsive genes were normalized to expression of *gapdh* for each cell line tested.

Jurkat, MT-1, ED40515 (-), 43 Tb (-), ED40515 (+), and LM-Y1 cells were untreated or treated with 2 μM or 5 μM 9AA for 48 hours. After treatment, cells were stained with antibodies that detected active caspase-3 and cleaved PARP. A minor double positive population of active caspase-3/cleaved PARP was detected in all the cell lines in the absence of 9AA (Figure 2; top panels). A two- to 20-fold increase in caspase-3/cleaved PARP double positive cells was seen in the five ATL cell lines depending on the concentration of 9AA used (Figure 2). In contrast, the percentage of caspase-3/cleaved PARP double positive Jurkat cells did not change after 9AA treatment (Figure 2). Consistent with the increase in caspase-3/7 activity in 9AA treated MT-1, 43 Tb (-), and ED40515 (-) cells (Figure 3B), western blot analysis showed increased PARP cleavage (Figure 3A). These changes were not detected in 9AA treated Jurkat cells (Figure 3A and B). These results indicate that 9AA induces apoptosis in ATL cells.

We have previously shown that 9AA inhibits the NF-κB pathway while activating the p53 signaling pathway [24]. To determine the impact of 9AA on the activation status of both p53 and NF-κB signaling in the ATL leukemic cells, MT-1, 43 Tb (-), and ED40515 (-) cells were treated with 9AA at 10 μM for 48 hours. Jurkat cells, which do not respond to 9AA treatment, were used as a control. After treatment, the protein level of p53 increased in MT-1 and 43 Tb (-) cells, but not in ED40515 (-) cells. ED40515 (-) cells have previously been shown to have mutant p53 with very low to undetectable protein levels [25]. The mutational status of p53 in MT-1, 43 Tb (-) and ED40515 (-) cells was confirmed by sequencing. Importantly, independent of the p53 status, phosphorylation of p65 decreased in 9AA treated MT-1, 43 Tb (-), and ED40515 (-).Similarly, we saw inhibition of NF- κB activation in all HTLV-1 infected cell lines. 9AA treatment did not affect the level of p65 protein in any of the cell lines but specifically in HTLV-1 infected lines 9AA reduced p65 phosphorylation, as well as phosphorylation of the IKKα/β kinases and the NF-κB inhibitor IκBα (Figure 3A). XIAP protein (an NF-κB responsive gene) was also reduced in HTLV-1 leukemic cells after treatment with 9AA. To note, inhibition of NF-κB in Jurkat cells which are resistant to 9AA was not detected (Figure 3A).

To determine if 9AA affected p53 transcriptional activity, we measured the level of the p53-responsive genes *hdm2* and *bax*. In 43 Tb (-) cells that have a wild type p53, we see induction of both *hdm2* and *bax* gene expression (Figure 3C). In ED40515 (-) and MT-1 cells, which carry a mutant p53 gene and the 9AA resistant cell line Jurkat, we see no significant induction of *hdm2* and only in MT-1 cells do we see a 2 fold induction of *bax*. These results indicate that 9AA can reactivate wild type p53 and inhibit p65 activation selectively inducing apoptosis in ATL cells.

### 9AA prolonged the survival period of MET-1 leukemia-bearing mice

**Figure 4:**
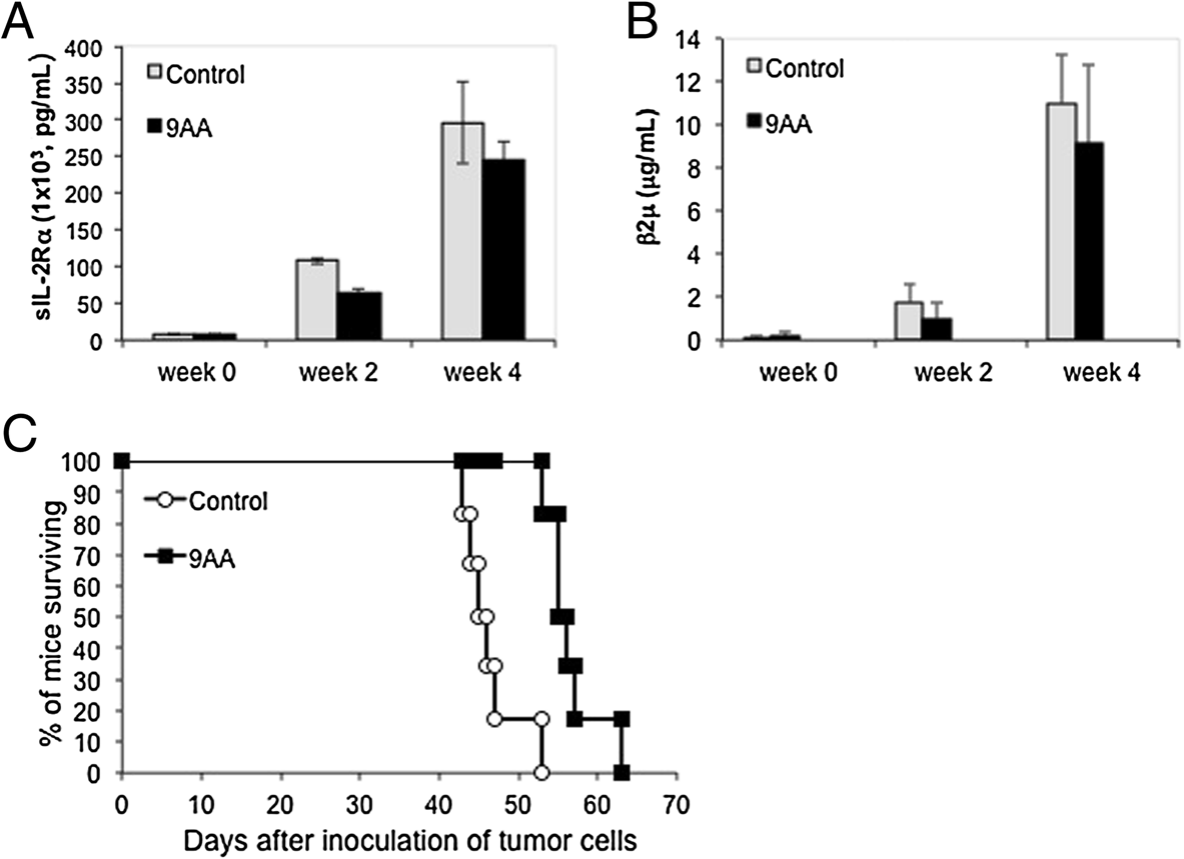
**9AA modestly prolonged the survival period of MET-1 leukemia-bearing mice. (A)** Serum levels of human soluble IL-2Rα (s IL-2Rα) during the course of treatment of NOD/SCID mice. At the beginning of the therapy, the serum levels of sIL-2Rα for the control group and 9AA group were 6,749 pg/mL and 7,223 pg/mL, respectively. Two weeks after therapy, the serum values of the control group and 9AA treated group were 107,606 pg/mL and 64,538 pg/mL, respectively (*p* = .11). Four weeks after therapy, the values of sIL-2Rα between the control group and the 9AA group were 295,636 pg/mL and 245,642 pg/mL, respectively (*p* = .17). **(B)** The mean serum β2μ levels during the course of treatment in NOD/SCID mice. At the beginning of the therapy, the serum levels of β2μ for the control group and 9AA group were 0.07 μg/mL and 0.17 μg/mL, respectively. Two weeks after therapy, the serum values of the control group and 9AA treated group were 1.76 μg/mL and 0.99 μg/mL, respectively (*p* = .11). Four weeks after therapy, the values of β2μ were 11 μg/mL and 9.2 μg/mL for the control group and the 9AA group, respectively (*p* = .32). **(C)** Kaplan-Meier analysis demonstrating 9AA prolonged the survival of mice bearing the MET-1 leukemia. The control group mice died between days 43 and 53, and its median survival duration was 45.5 days. The 9AA group mice died between days 53 and 63, and its median survival duration was 55.5 days. Compared to the control group, there was a significant prolongation of the survival of the mice treated with 9AA (*p* = .0056).

As discussed earlier, the majority of primary ATL cells express wild type p53 in an inactive state and constitutive NF-κB activation. To evaluate the therapeutic capacity of 9AA in ATL disease, we use an acute human ATL (MET-1) xenograft murine model. The MET-1 ATL cells in this model are activated T-cells that have a distinct phenotype as characterized by FACS analysis: CD2^+^, CD3^dim^, CD4^+/-^, CD7^-^, CD20^-^, CD122, and CD25^+^. They also express high levels of CD52, the target of the Campath-1H (alemtuzumab) monoclonal antibody [26]. At the beginning of the therapy, the average serum levels of soluble IL-2Rα, a surrogate tumor marker in this murine model, for the control group and 9AA group were 6748.5 pg/mL and 7223 pg/mL, respectively. A 2-week course of treatment with 9AA (150 μg/mouse/day, a 14-day infusion) demonstrated modest but statistically significant therapeutic efficacy as assessed by prolonged survival of leukemia-bearing mice (*p* = .0056, Figure 4C). Two surrogate tumor markers in the MET-1 model, sIL-2Rα and β2μ, showed a modest reduction in serum levels of 9AA treated mice (Figure 4A and B). Based on these observations and the reasoning that p53 reactivation may need an additional assault to become fully clinically effective, we moved our studies to a further therapeutic trial by using 9AA in combination with another immunotherapeutic reagent.

### 9AA combined with Campath-1H significantly prolonged the survival period of MET-1 leukemia-bearing mice

Because we demonstrated previously that the humanized anti-CD52 antibody, Campath-1H, which acts by antibody directed cellular cytotoxicity (ADCC), had immunotherapeutic activity in the MET-1 xenograft mouse model and in ATL patients [27,28], we hypothesized that the combination of 9AA with Campath-1H might be a rational new combination therapy for ATL patients. To investigate this, we conducted a therapeutic trial with 9AA alone, Campath-1H alone, and the combination of 9AA with Campath-1H in the MET-1 model of human ATL.

**Figure 5:**
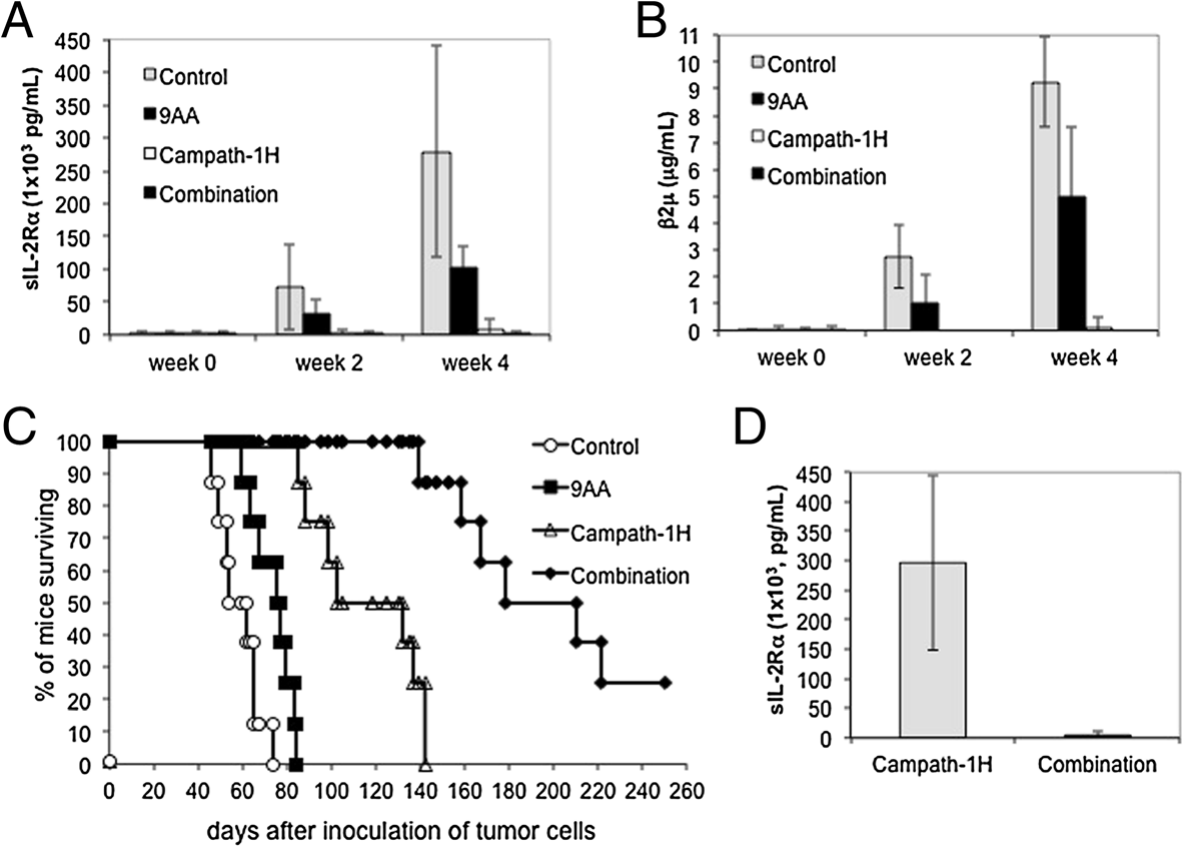
**9AA combined with Campath-1H significantly prolonged survival of MET-1 leukemia-bearing mice. (A)** On day 1, the serum levels of sIL-2Rα for the four groups ranged from 3,189 to 3,486 pg/mL. Two weeks after therapy, the serum values rose to 72,737 pg/mL for control mice and 32, 654 pg/ml for 9AA mice (9AA *vs* control, *p* > .05). The sIL-2Rα levels for Campath-1H (Campath-1) treated mice showed no increase compared to initial levels, 3,270 pg/mL (Campath-1H *vs* control, *p* < .01). The combination group decreased to 1,810 pg/mL (combination *vs* control, or *vs* 9AA, *p* < .01). Four weeks after therapy, sIL-2Rα was 279,302 pg/mL and 102,233 pg/mL for control and 9AA groups, respectively (*p* < .01). Serum sIL-2Rα for the Campath-1H group increased to 7,674 pg/mL (Campath-1H *vs* control, *p* < .001). The combination group remained at 1,330 pg/mL (combination *vs* control, or *vs* 9AA, *p* < .001). **(B)** On day 1, the serum levels of β2μ for the four groups were less than 0.05 μg/mL. Four weeks after therapy, the serum β2μ values of the control and 9AA groups were 9.25 μg/mL and 5.0 μg/mL, respectively (*p* > .05). The serum β2μ values of the Campath-1H group increased to 0.13 μg/mL (Campath-1H *vs* control, *p* < .0001). The serum β2μ values were below detection for the combination group (combination *vs* control, or *vs* 9AA, *p* < .0001). **(C)** Kaplan-Meier analysis demonstrating combination therapy of 9AA and Campath-1H prolonged survival of MET-1 leukemia-bearing mice. (*p* < .0001). **(D)** Four of 8 mice in the Campath-1H group and 8 of 8 mice in the combination group survived to 100 days. The sIL-2Rα levels for the groups of Campath-1H and the combination were 296,467 pg/mL and 4,609 pg/mL, respectively (*p* < .001).

A 2-week course of treatment with 9AA (150 μg/mouse/day, a 14-day infusion), a 4-week course of treatment with Campath-1H (100 μg/mouse, weekly), and the combination of 9AA and Campath-1H manifested therapeutic efficacy as assessed by demonstration both of reduced serum levels of sIL-2Rα and β2μ and prolongation of survival of the leukemia-bearing mice (Figure 5). When compared with the PEG300 control group of mice, at week 2 and week 4, there was a significant reduction of serum levels of sIL-2Rα in the groups treated with Campath-1H alone (*p* < .01), and the combination of 9AA with Campath-1H (*p* < .01, Figure 5A) as well as a reduction in serum β2μ levels at week 2 and week 4 in the Campath-1H alone group (*p* < .0001) and in the combination group ( *p* < .0001, Figure 5B). Furthermore, the mean sIL-2Rα levels were significantly lower in the combination group 100 days post-therapy when compared with that of the group that received Campath-1H alone (*p* < .001, Figure 5D).

The mice in the PEG300 control group died between day 46 and day 74 with a median survival of 58 days. The 9AA treatment alone, the Campath-1H treatment alone, and the combination treatment significantly prolonged the survival of leukemia-bearing mice (Figure 5C). The mice in the 9AA treatment group died between day 59 and day 84 with a median survival of 76 days (*p* < .01), comparable to the control group. The mice in Campath-1H alone treatment group died between day 85 and day 142 with median survival duration of 100 days (*p* < .0001). Compared with both the 9AA alone and the Campath-1H alone groups, there was a significant prolongation of the survival of the mice treated with the combination of 9AA and Campath-1H with median survival duration of 194 days (*p* < .0001) (Figure 5C). Two of 8 mice in the combination therapy group survived more than 250 days.

### Induction of p53 and apoptosis in splenic cells from leukemia-bearing mice

**Figure 6:**
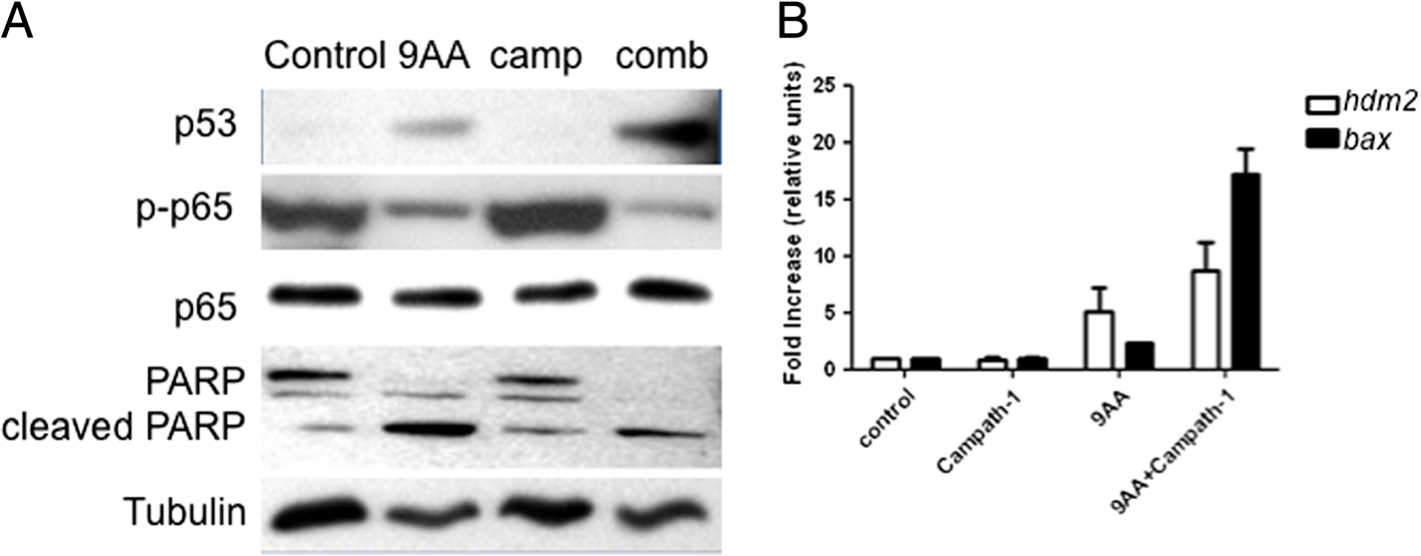
**Induction of p53 *ex vivo *in 9AA and 9AA with Campath-1H treated MET-1 cells. (A)** Western blot analysis of expression levels of p53, phospho-p65, p65, and PARP in splenocytes from MET-1 injected mice. Thirteen days after therapy, splenic cells from MET-1 tumor-bearing mice were separated. Cell lysates were immunoblotted with the indicated antibodies: PARP, p53, phospho-p65 and p65. α-Tubulin was used as an input control. **(B)** p53 responsive genes increased after 9AA and 9AA plus Campath-1H treatment. Total cellular RNA was isolated from splenic cells of mice from each treatment group. RT-PCR was performed and the levels of *hdm2* and *bax* genes were measured. The levels of *hdm2* and *bax* for control animals were set at 1 and the graph represents results from three independent experiments.

Next we collected spleens from mice in each study group to evaluate p53. The expression of p53 was very weak in lysates from splenic cells of untreated or Campath-1H treated MET-1 leukemia-bearing mice. Treating mice with 9AA alone enhanced the p53 expression, while the combination of 9AA with Campath-1H profoundly increased the expression of p53. Similarly, 9AA alone or in combination with Campath-1H decreased phosphorylation of p65 (Figure 6A). The overall p65 protein levels did not change, indicating that 9AA and 9AA plus Campath-1H inhibited NF-κB activation.

Consistent with decreased tumor burden, treatment with 9AA alone increased the level of PARP cleavage, but the combination treatment resulted in more pronounced cleavage of full length PARP (Figure 6A). To assess p53 function, total RNA was isolated from splenic cells and the level two p53 responsive genes *hdm2* and *bax* were measured by RT-PCR. Compared to control (untreated) levels, treatment with 9AA alone resulted in a 5-fold induction of *hdm2* mRNA and a 2-fold induction of *bax* mRNA (Figure 6B). However, mice treated with the combination of 9AA and Campath-1H had a greater induction of both *hdm2* (8-fold) and *bax* (16-fold) mRNA levels, consistent with the increased efficacy of the combination treatment (Figure 6B). Treatment with Campath-1H alone did not increase expression of either *bax* or *hdm2* above control samples.

## Discussion

Adult T-cell leukaemia/lymphoma is an aggressive T-cell malignancy that occurs in 2-4% of HTLV-1 infected individuals. Treatment of ATL includes antiviral drug therapy, conventional chemotherapy, monoclonal antibodies and in some cases transplantation, however, responses to therapy are poor and not long-lasting [29]. Thus new approaches to treatment are required. Our data shows that 9AA has an antiproliferative effect both *in vitro* and *in vivo*. In leukemic cells, 10 μM 9AA caused an approximately 60% drop in proliferation after 24 hours of treatment. Further, 9AA treatment induced increased p53 protein levels in cells carrying wild type p53. However, independent of p53 status, 9AA induced caspase-3/7 activity selectively in HTLV-1-infected ATL cell lines and inhibited NF-κB activation.

A preclinical *in vivo* murine model of ATL developed in our lab has been successfully used to test new therapeutic agents [26,30-33]. When tested in mice, 9AA did not show toxicity and alone had only a modest effect on tumor burden and survival. In our murine model, we have often seen that the combination of a monoclonal antibody with other chemotherapeutic agents can afford synergistic effects [32,33]. Previously, we showed that anti-CD52 antibody acts by antibody directed cellular cytotoxicity (ADCC) and has immunotherapeutic activity in the MET-1 xenograft mouse model and in ATL patients [27,28]. In the present study, we tested the combination therapy of 9AA with Campath-1H. Together 9AA and Campath-1H had a significant synergistic therapeutic effect in the MET-1 murine model of ATL. Of note 2 of the 8 mice in the combined treatment group survived for the duration of the experiment (250 days). In splenic samples from drug treated MET-1 bearing mice, we show that 9AA alone or in combination with Campath-1H reactivates p53 causing apoptotic cell death. These results suggest that 9AA reactivation of p53 in tumor cells is insufficient but may require a second hit that is associated with tumor cell death to be truly effective as a therapeutic agent. Indeed, when the combination therapy is used in our model, a dramatic increase in Bax, a strong proapoptotic inducer, is seen above that observed with 9AA treatment alone.

In light of the important role p53 plays in maintaining genomic stability, it is a key therapeutic target. p53 functions as an integrator of stress response signals by activating or repressing the transcription of genes that regulate cell cycle progression, growth arrest, senescence and/or apoptosis [7,34-38]. The importance of p53 function is underscored by the observation that it is the most commonly mutated gene in human tumors [6]. However, the frequency of p53 mutations in untreated ATL patients is low compared to other human cancers [14,16,39-41]. We and others have previously shown that p53 is functionally inactivated in HTLV-1 infected and ATL cells *in vitro* and *ex vivo*[11,17,39]. We went on to demonstrate that Tax activation of the NF-κB pathway involving p65 phosphorylation and IKKβ kinase activity was linked to p53 inactivation [18-21]. This was demonstrated using knockout mouse embryo fibroblasts, p65 mutant constructs as well as siRNA knockout of p53 and IKKβ [19,20].

9AA was initially identified as an antibacterial agent [42,43] and later found to interact with guanidinobenzoatases and that it could be used to locate malignant cells in many tumor tissues [44]. Studies by Wang et al. [45] demonstrated that in a non-small cell lung carcinoma cell line, an ovarian cancer cell line, and a colon adenocarcinoma cell line, that the acridine derivatives 9AA, amsacrine, quinacrine and acridine orange, stabilized p53 and induced p53 dependent apoptosis. They further showed that knockout of Bax, a p53 target and a key cell death inducer, blocked acridine derivatives from inducing cell death. Corroborating our work on HTLV-1, Gurova and colleagues went on to show that 9AA targets two important stress-responsive pathways, NF-κB and p53, in Renal cell carcinoma (RCC) cell lines, as well as the prosurvival AKT/mammalian target of rapamycin (mTOR) pathway [22,24,46].

More recent studies indicate that the acridine derivative, quinacrine that has been used in the treatment of malaria, giardiasis, and rheumatic disease, stabilizes p53 and induces p53-dependent and p53- independent cell death [45,47]. El-Deiry’s group evaluated the potential of adding quinacrine to anticancer chemotherapeutics and targeted agents as a potential novel combinatorial therapy for advanced colon cancer. Using a panel of 10 human colorectal cancer cell lines, quinacrine was shown to synergize with 5-fluorouracil (5-FU) and significantly enhance the cytotoxicity of sorafenib. In addition, they found that while quinacrine alone lowered the tumor load of nu/nu mice bearing human colorectal cancer xenografts, the combination of quinacrine and 5-FU caused a significant delay in tumor growth of a variety of different xenografts when compared to each agent administered alone [48]. In addition, quinacrine rendered resistant hepatocellular carcinoma cells sensitive to treatment by TRAIL, inducing overwhelming cell death within hours. These results suggest that using quinacrine in combination with chemotherapeutic agents and targeted agents could improve treatment of patients with recurrent, locally advanced, or metastatic colorectal cancer [47].

In recent studies Gurova’s group sought to identify more potent molecules with similar properties to quinacrine [49]. This led to isolation of curaxins, a distinct structural class of compounds with similar effects on p53 and NF-κB that lack genotoxicity but maintain tumor cell–specific cytotoxicity. The authors indicate that the effects of curaxins on p53 and NF-κB, as well as their toxicity to cancer cells, result from “chromatin trapping” of the FACT (facilitates chromatin transcription) complex and casein kinase 2 phosphorylation of p53 at serine 392. The modulation of p53 and NF-κB activities by curaxins suggests that they have strong potential for development into effective anticancer drugs [49].

The p53 and NF-κB pathways are dysregulated in nearly all tumors, making them attractive targets for therapeutic agents. We have identified that 9AA is effective in reactivating p53 and inhibiting p65 phosphorylation in ATL leukemic cell lines. Interestingly, even in HTLV-1 infected ATL cells which carry a mutant p53, apoptotic cell death was observed. Based on work by several groups which demonstrated that inhibitors of NF-κB induce apoptosis in HTLV-1 transformed cells [50-54] we speculate that the 9AA induced inhibition of NF-κB that we found in leukemic cells carrying mutant p53 is sufficient to induce apoptosis. Further, we found that 9AA reduced the tumor burden in our NOD/SCID, MET-1 ATL mouse model. A more profound effect was found using 9AA in combination with Campath-1H. Combination therapy significantly delayed leukemic cell growth through robust activation of p53 and inhibition of NF-κB. These results are consistent with those found for hepatocellular carcinoma and colorectal cancer where a synergistic effect was seen between a quinacrine derivative and a second anti-tumor agent [47,48]. Together, our results suggest that further investigation into compounds which reactivate p53 function in combination with current chemotherapies or targeted therapies should be pursued for the treatment of ATL.

## Conclusions

In summary, these studies demonstrate that 9-aminoacridine selectively induces cell death in ATL leukemic cells through inhibition of NF-κB activation and induction of p53 responsive genes. The combination of Campath-1H and 9AA significantly enhanced the survival of MET-1 tumor bearing mice when compared to each agent administered alone. Thus, these findings suggest that acridine-derivatives in combination with other chemotherapeutics could improve treatment of ATL.

## Methods

### Compound and other materials

9-aminoacridine (9AA) was from Sigma-Aldrich (St. Louis, MO). For *in vitro* studies, stock solutions of 9AA were made in dimethylsulfoxide (DMSO), and subsequently diluted in RPMI 1640 medium for use. For *in vivo* studies, 9AA was dissolved in a solution with 10% polyethylene glycol 300 (PEG300) (VWR, Bridgeport, NJ) and continuously administrated via a subcutaneous mini-osmotic pump (Alzet, Cupertino, CA). Campath-1H was from Genzyme Corporation (Cambridge, MA) and diluted in PBS before use.

### Analysis of p53 sequence

Total RNA from MT-1, 43 Tb (-) and ED40515 (-) cell lines was reverse transcribed using a SuperScript IIII First-Strand Synthesis System (Life Technologies). Oligonucleotide primers used for the PCR amplification of the p53 gene are as follows: p53F1 5′-TCCGGGGACACTTTGCGTT-3′, p53F2 5′-ACGCCAACTCTCTCTAGCTC-3′ and p53R1 5′-GGTGCTTCTGACGCACACCT-3′. PCR amplification was carried out using the Phusion High Fidelity Kit (Thermo Scientific) according to the manufacturer’s instructions. The parameters for the PCR reaction was 98°C for 2 min, followed by 35 cycles of denaturation at 98°C for 10 s, annealing 68°C for 1.5 min, and extension at 72°C for 30 s. The extension time of the last cycle was 72°C for 10 min. The amplified PCR product was purified using QuickStep 2 PCR Purification Kit (Edge Bio). The amplified PCR products were sequenced directly using the BigDye Terminator v1.1 Cycle Sequencing Kit (Applied Biosystems) and the oligonucleotide primers used in the PCR amplification. We confirmed that p53 in MT-1 cells has a missense mutation in exon 5 [55], is undetectable at both the mRNA and protein levels in ED40515 (-) [25] and wild type in 43 Tb (-) cells.

### Cell proliferation assay

HTLV-1-infected, cytokine independent leukemic cell lines MT-1, ED40515 (-), and 43 Tb (-) were cultured in RPMI 1640 medium. HTLV-1-infected, cytokine dependent leukemic cell lines ED40515 (+) and LM-Y1 were cultured in RPMI 1640 medium supplemented with 100 units of interleukin-2. The five HTLV-1-infected leukemic cell lines are truly derived from the leukemic cells of the patients as demonstrated by analysis of TCR rearrangement [56]. LM-Y1, 43 Tb (-) and MET-1 have wild-type p53; MT-1 and ED40515 (-) have mutant p53. Aliquots of 1.5×10^4^ cells/100 μl/well cells were seeded into 96-well plates with or without 9AA and incubated at 37°C, 5% CO_2_ in an incubator for 24, or 48, or 72 hours. The T-cell leukemia cell line-Jurkat or an acute lymphoblastic leukemia cell line-CEM was used as controls. 1 μCi of ^3^H-thymidine (PerkinElmer, Waltham, MA) was added into the cells for the last 6 hours. After incubation all cells were harvested and counted with a β counter. The assay was performed in triplicate in 3 independent experiments.

### Detection of cleaved PARP and active caspase-3

Jurkat, MT-1, ED40515 (-), 43 Tb (-), ED40515 (+), and LM-Y1 cells were treated with or without 9AA for 48 hours. After treatment, the cells were washed with cold PBS, and fixed with 2% paraformaldehyde for10 minutes at room temperature. The cells were washed with FACS buffer, then permeabilized and co-stained with anti-cleaved PARP and anti-active caspase-3 (BD Pharmingen, San Jose, CA) antibodies diluted in FACS buffer containing 0.25% saponin (Sigma-Aldrich) for 30 minutes at room temperature. After two washes with FACS buffer containing 0.25% saponin, the cells were suspended in FACS buffer and analyzed by Flow cytometry. The assay was performed in triplicate in 3 independent experiments.

### Immunoblotting

Jurkat, MT-1, 43 Tb (-), and ED40515 (-) cells were treated with or without 10 μM of 9AA for 48 hours. Cells were solubilized at 4°C in lysis buffer. The cells lysates (50 μg) were resolved by electrophoresis in 4 to 12% SDS-PAGE gels and transferred to PVDF membranes (Invitrogen, Grand Island, NY). After blocking, the blots were incubated with the indicated antibodies: PARP, phospho-p65 (p-p65), p65, XIAP, IκBα, phospho-IκBα, phospho-IKKα\β and β-actin (Cell Signaling Technology, Danvers, MA), p53 (BioLegend, San Diego, CA), or tubulin (Sigma-Aldrich). The splenic cells from tumor bearing mice were separated 13 days after therapy was completed and checked for their status of p53, PARP, p-p65 and p65.

### Caspase-3/7 activity assay

Cells were plated in white-walled 96-well tissue culture plates at a density of 2×10^4^ cells per well in 100 μl of medium. Jurkat, MT-1, 43 Tb (-), and ED40515 (-) cells were treated in triplicate with or without 10 μM of 9AA for 48 hours. Caspase-3 and -7 activity was determined using the Caspase-Glo®3/7 assay kit (Promega, Madison, WI) as directed by the manufacturer. Briefly, Caspase-Glo®3/7 reagent was added to each well in a 1:1 ratio and incubated for 1 hour at room temperature before measuring luminescence. The activity of caspase-3/7 was normalized to that of the untreated sample under the same plating and density conditions.

### Therapeutic studies of 9AA alone and in combination with Campath-1H in MET-1 human ATL leukemia-bearing mice

The human ATL cell population, MET-1, was established from the peripheral blood of a patient with acute ATL and were maintained by serial transfer in NOD/SCID mice (Jackson Laboratories, Bar Harbor, ME) as described previously [30]. The MET-1 leukemia model was performed by intraperitoneal injection of 1.5 × 10^7^ MET-1 cells in NOD/SCID mice. Therapeutic trials were performed on these mice when their serum soluble IL-2Rα (sIL-2Rα) levels ranged from 1000 to 10,000 pg/mL, approximately 10 to 14 days after inoculation of tumor cells. All animal experiments were performed in accordance with National Institutes of Health Animal Care and Use Committee guidelines and were approved by this committee. There were three groups of mice with 6 mice per group in the 9AA therapeutic trials. The mice were randomly assigned to groups and had comparable mean levels of the surrogate tumor marker, sIL-2Rα, at the beginning of the experiments. Groups 1 and 2 received an inoculation of MET-1 cells. Group 1 received vehicle PEG300 by subcutaneous-osmotic pump infusion for 14 days. Group 2 received 9AA at 150 μg/mouse/day for 14 days by subcutaneous-osmotic pump infusion. Group 3, with no tumor and no therapy, served as a control for the natural survival of NOD/SCID mice in the animal facility. For the further combination therapeutic study of 9AA with Campath-1H, there were five groups of mice with 8 mice per group in the therapeutic trials. Groups 1 to 4 received an inoculation of MET-1 cells. Group 1, the control group, received vehicle PEG300 by subcutaneous-osmotic pump infusion for 14 days. Group 2 received 9AA alone by subcutaneous-osmotic pump infusion at 150 μg/mouse/day for 14 days. Group 3, Campath-1H (humanized anti-CD52, alemtuzumab) alone, received the Campath-1H by intravenous injection of 100 μg on days 0, 7, 14, and 21. Group 4, the combination therapy group, received 9AA and Campath-1H as described in group 2 plus group 3. Group 5, with no tumor and no therapy, served as a control for the natural survival of NOD/SCID mice.

### Monitor of tumor growth

Measurements of the serum concentrations of human sIL-2Rα and soluble human β-2-microglobulin (β2μ) were performed using enzyme-linked immunosorbent assays (ELISA) as indicated in by the manufacturer (R&D Systems, Minneapolis, MN).

### Reverse transcription-PCR (RT-PCR)

Total RNA was prepared with Trizol reagent (Invitrogen) using the manufacturer’s protocol. One microgram of total cellular RNA was reverse transcribed with the QuanTitect reverse transcription kit (Qiagen, Valencia, CA). cDNA quantification for GAPDH, HDM2 and Bax was performed by real-time PCR (ABI 7000). The primers used were: gapdh: 5′- GGT CTC CTC CGA CTT CAA CA-3′ (forward) and 5′- TGC TGT AGC CAA ATT CGT TG-3′ (reverse); HDM2: 5′- GGT TGA CTC AGC TTT TCC TCT TG-3′ (forward) and 5′- GGA AAA TGC ATG GTT TAA ATA GCC-3′ (reverse). The PCRs contained 1× SYBR Green mix (Stratagene, Santa Clara, CA), a 400 nM concentration of each primer, and 50 ng of cDNAs.

### Statistical analysis

Serum levels of human sIL-2Rα and β2μ were analyzed at different time points among different treatment groups using the Student’s *t* test for unpaired data. The statistical significance of differences in survival of mice in different groups was determined by the log-rank test using the StatView program (Abacus Concepts, Berkeley, CA).

## Abbreviations

ATL: Adult T-cell leukemia/lymphoma; HTLV-1: Human T-cell leukemia virus type 1; 9AA: 9-aminoacridine; β2μ: β2-microglobulin; sIL-2Rα: Soluble interleukin-2 receptor alpha; ADCC: Antibody directed cellular cytotoxicity; FACT: Facilitates chromatin transcription.

## Competing interests

The authors declare that they have no competing interests.

## Authors’ contributions

WJ participated in the design and performed the research, analyzed the data, and wrote the manuscript; CPM participated in the design and performed the research, and wrote the manuscript; MZ participated in the design of the research and performed experiments; MP participated in the design of the research and performed experiments; MM developed the ATL cell lines; TAW designed the research and wrote the manuscript; all authors reviewed and approved the manuscript.
